# Therapeutic Efficacy of Kangfuxin Liquid Combined with PPIs in Gastric Ulcer

**DOI:** 10.1155/2019/1324969

**Published:** 2019-09-30

**Authors:** Jun-Bo Zou, Xiao-Fei Zhang, Ya-Jun Shi, Jia Tai, Yu Wang, Yu-Lin Liang, Fang Wang, Jiang-Xue Cheng, Jing Wang, Dong-Yan Guo

**Affiliations:** ^1^Shaanxi Province Key Laboratory of New Drugs and Chinese Medicine Foundation Research; Pharmacy College, Shaanxi University of Chinese Medicine, Xianyang, China; ^2^Key Laboratory of Modern Prepararation of Traditional Chinese Medicine, Ministry of Education, Jiangxi University of Traditional Chinese Medicine, Nanchang, China

## Abstract

**Objective:**

To evaluate the clinical efficacy and safety of Kangfuxin liquid (KFX) combined with proton pump inhibitors (PPIs) in the treatment of gastric ulcer (GU).

**Materials and Methods:**

Electronic databases including PubMed, Wanfang, CNKI, VIP, Embase, Cochrane Library, and CBM were examined for appropriate articles without language limitations on key words before March 10, 2019. RevMan 5.3 software was applied to execute outcome assessment and finish the meta-analysis.

**Results:**

22 articles involving 2,024 patients with a gastric ulcer were selected. Total efficacy rate and efficacy rate of gastroscopy were significantly enhanced for the combination of KFX with PPIs compared to those of PPI treatment alone (OR = 6.95, 95% CI: 4.87, 9.91, *P* < 0.00001; OR = 2.96, 95% CI: 1.98, 4.42, *P* < 0.00001, respectively). Same results were found for different PPIs in combination on total efficacy rate, respectively. The combination also significantly reduced the adverse events (OR = 0.39, 95% CI: 0.22, 0.70, *P*=0.002). In addition, KFX combined with PPI could suppress the inflammation (MD = −6.11, 95% CI: −7.45, −4.77, *P* < 0.00001), reduce the recurrence rate (OR = 0.31, 95% CI: 0.14, 0.70, *P*=0.005), and enhance the clearance rate of *Helicobacter pylori* (HP, OR = 3.76, 95% CI: 1.80, 7.87, *P*=0.0004). It seemed like the combination would influence immune function by increasing levels of T-lymphocyte subsets CD4 and CD8 but not CD3 (MD = 2.40, 95% CI: 1.25, 3.55, *P* < 0.0001); MD = 25.72, 95% CI: 14.55, 36.90, *P* < 0.00001; MD = 0.72, 95% CI: −0.66, 2.09, *P*=0.31, respectively).

**Conclusion:**

KFX combined with PPIs in treatment of patients with GU could improve the total efficacy rate and efficacy rate of gastroscopy and reduce adverse events and the recurrence rate. However, the results of this study should be handled with care due to the limitations. Several rigorous RCTs are in need to confirm these findings.

## 1. Introduction

Peptic ulcer (PU) is a common and prevalent disease worldwide. Infection of *Helicobacter pylori* (HP), nonsteroidal anti-inflammatory drugs (NSAIDs), and aspirin drug usage are considered to be the major causative factors of PU. Taking other medications including glucocorticoids, some antitumor drugs, and anticoagulant drugs is also inducements for PU, which cannot be neglected [1]. Prevalence rates of PU reach up to 5%–10% in 2009 [2] and continue to rise due to unhealthy lifestyle, drug usage, and diet custom. Gastric ulcer (GU) is one of the common types appearing mostly in middle-aged and elderly people [3].

Suppressing gastric acid production is the most important measure to relieve clinical symptoms and promote healing. Proton pump inhibitors (PPIs) are the initial therapy for GU [4]. The first generation PPIs includes omeprazole, pantoprazole, and lansoprazole. The second generation PPIs including esomeprazole, ilaprazole, and rabeprazole have faster onset time, longer action time, and fewer side effects. PPIs are the best drugs for treating acid-related diseases in the last dozen years. The medication amount of PPIs in domestic hospitals of 16 key cities in China reached 4.5 billion yuan in 2016 with an increase of 6% over the previous year, and it still presents a rising trend [5]. But long-term use of PPIs can cause a series of new safety issues, such as adverse renal effects [6], hypomagnesaemia [7], increased risk of dementia [8], increased risk of infection and osteoporosis [9], fracture risk [10], vitamin B12 deficiency, occurrence or development of atrophic gastritis, interstitial nephritis, microscopic colitis, increased risk of serious skin allergy, and so on [11, 12]. Combination with traditional Chinese medicine provides an alternative to improve the current situation of PPI usage.

**Figure 1:**
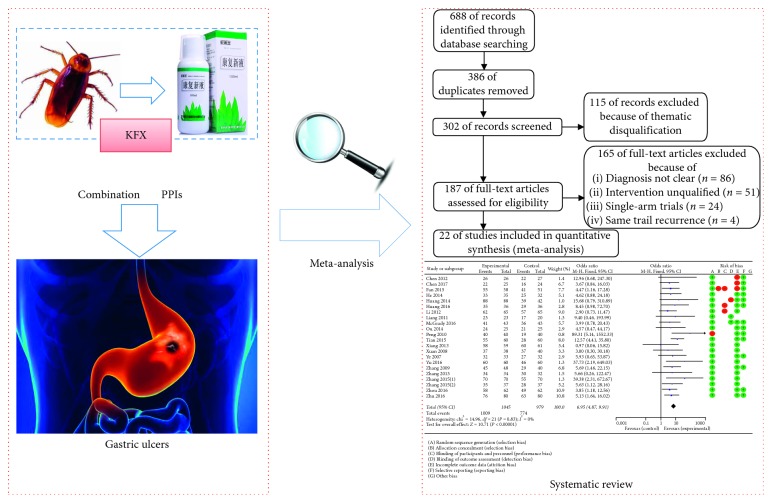
Work flow of the present study.

Kangfuxin liquid (KFX) is a Chinese patent medicine extracted from *Periplaneta americana.* China Food and Drug Administration (CFDA) approved it in 1998. As an animal medicine, amino acids are considered as the main ingredients which are used as quality control for preparation. Efficacy of KFX is described as promoting blood circulation, nourishing yin, and promoting granulation. For oral administration, it is used for gore block, stomach bleeding, gastric and duodenal ulcers, phthisis with yin deficiency, and aiding in the treatment of tuberculosis. For external application, it is used for treating incised wound, trauma, ulcers, fistula, burns, and bedsore. Amounts of clinical trials have demonstrated that KFX is beneficial to cure GU [13–15], which is also supported by animal experiments [16]. Previous meta-analysis confirmed that KFX combined with PPIs was superior to PPIs alone in the treatment of GU [17, 18]. We provide an updated and extended meta-analysis with detailed information for efficacy (Figure 1).

## 2. Methods and Program

### 2.1. Literature Retrieval Strategy

Keywords “kangfuxin (KFX)” [Title/Abstract] OR “*Periplaneta americana*” [Title/Abstract] AND “Gastric ulcer” [Title/Abstract] OR “peptic ulcer” [Title/Abstract] OR “digestive ulcer” [Title/Abstract] OR “PPI” (including Esomeprazole, Omeprazole, Lansoprazole, Rabeprazole, Pantoprazole) [Title/Abstract] were used as search items in electronic databases including PubMed, Wanfang, CNKI, VIP, Embase, Cochrane Library, and CBM. Articles published before March 10, 2019, was examined without language limitations in order to obtain a comprehensive retrieval. All relevant articles were downloaded into EndNote software (version X7, Thomson Reuters, Inc., New York, USA) for further exploration. Duplicate records were integrated. Full-text review was performed while the title/abstract was thought to be thematic. The job above was executed by two investigators independently. Conflicts were resolved by consensus and discussion.

### 2.2. Inclusion and Exclusion Criteria

Based on the suggestions of a gastroenterologist, we designed the inclusion criteria as follows: (1) Patients in RCTs diagnosed as having gastric ulcers by meeting the criteria of Diagnosis and Treatment of Digestive Ulcer Disease (DTDUD) version 2016, 2013, 2008, or Practical Clinical Diagnosis and Treatment of Digestive Disease (PCDTDD, part 1) version 2005, or the Guiding Principle of Clinical Research of new TCM on the Treatment of Peptic Ulcer (GPCRTPU), or Diagnostics of Digestive Diseases (DDD) version 2006 [19], or carrying out gastroscopy. (2) All trials mentioned were confined as RCTs. (3) Patients in treatment groups received KFX solution combined with PPI while control groups received PPI alone. (4) The total efficacy rate was the least outcome measurement to be reported.

We also designed the exclusion criteria as follows: (1) References such as reviews, case reports, animal experiments, comments, and so on that are thought to be athematic. (2) The diagnostic standard in the statement was ambiguous. (3) Trials emphasized on other peptic ulcers but not gastric ulcer. (4) Trials mentioned other interventions of essential treatment to GU but not only PPI alone.

### 2.3. Characteristics of Study Assessment

Information including methods, participants, interventions, and outcomes was extracted and arranged (Tables 1 and 2). Characteristics of included studies were assessed by two searchers independently according to the Cochrane Handbook for Systematic Reviews of Interventions [41]. Disagreement was resolved by the consensus. Risk of bias was evaluated as follows: random sequence generation (A, selection bias), allocation concealment (B, selection bias), blinding of participants and personnel (C, performance bias), blinding of outcome assessment (D, detection bias), incomplete outcome data (E, attrition bias), selective reporting (F, reporting bias), and other biases (G). Three levels were applied to judge the quality of each item. “Low risk” indicates description of methods or procedures was adequate while “high risk” means not adequate or incorrect and “unclear risk” means missing description.

### 2.4. Data Analysis

Data analysis was performed using Review Manager 5.3 (Cochrane Collaboration). Outcome indices such as total efficacy rate and efficacy rate of gastroscopy were regarded as dichotomous variables and presented as the odds ratio (OR) with 95% confidence intervals (95% CI). Levels of inflammatory cytokines such as IL-6, TNF-*α*, and TGF-*β*1 and T-lymphocyte subsets including CD3, CD4, and CD8 were continuous variables which were presented as the mean difference (MD) with 95% CI. *Q* statistic and *I*^2^ tests were applied to assess the heterogeneity among studies. A fixed-effects model was used to analyze data with low heterogeneity (*P* > 0.1 and *I*^2^ ≤ 50%), while a random-effects model was used to analyze data with high heterogeneity (*P* < 0.1 or *I*^2^ > 50%). Potential publication bias was revealed by funnel plots.

## 3. Results

### 3.1. Characteristics of the Eligible Studies

**Figure 2:**
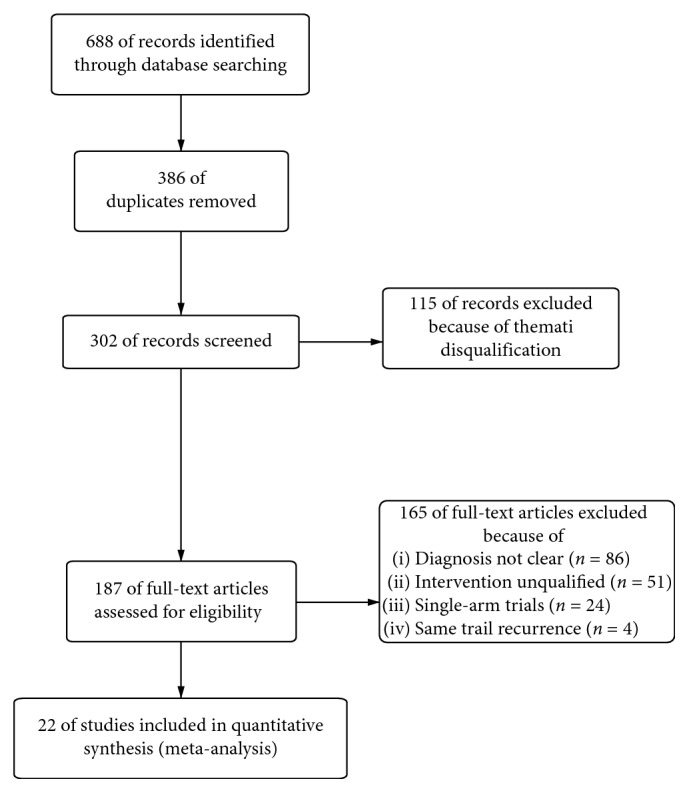
Process of the study extracted for the meta-analysis.

Six hundred eighty-eight articles were identified through database searching, in which 386 articles were removed as duplicates. 115 articles in 302 remaining were excluded on thematic disqualification. Then, 187 articles remained for further full-text review. 165 studies were excluded in this procedure for the following reasons: diagnosis in 86 articles was vague, 51 studies mentioned unfit interventions, 24 studies were single-arm designs, and 4 articles were the recurrence of the same trial. 22 studies [20–40, 42] were included in quantitative synthesis finally (Figure 2).

Two thousand twenty-four patients with a gastric ulcer (1045 cases in the experimental group and 979 cases in the control group) were taken in this meta-analysis. The age of the patients ranged from 17 to 75 years, and there was no obvious difference in terms of age and sex between the two groups (Table 1). Trials were conducted between 2007 and 2017, and all were RCTs with a comparison between a combination of KFX solution and PPI and PPI treatment alone. 4 studies [20, 21, 22, 23] reported the combination with esomeprazole, 5 with omeprazole [24–27, 39], 3 with lansoprazole [28–30], 3 with rabeprazole [31–33], and 7 with pantoprazole [34–38, 40, 42]. The treatment duration ranged from 1 to 4 weeks, and 2 articles [24, 40] reported a follow-up which ranged from half a year to 1 year. 10 trials [22, 26, 27, 29, 34, 36, 38–40, 42] reported adverse events and side effects. All trials reported a total efficacy rate in outcome measures, 15 studies [20–24, 27–32, 34, 35, 37, 38] reported the efficacy rate of gastroscopy, 3 studies [25, 34, 39] reported the clearance of HP, 2 studies [24, 39] reported the recurrence rate, and 2 trials [29, 34] reported the levels of inflammatory cytokines and T-lymphocyte subsets (Table 2).

### 3.2. Quality of Included Trials Assessment

According to the Cochrane risk of bias estimation, all trials mentioned a randomized allocation of participants while 1 trial used a wrong method, so the selection bias (A) on random sequence generation was considered to be “low risk.” Detailed information on allocation concealment, blinding of participants and personnel, and blinding of outcome assessment of all studies was ambiguous even wrong, from which the selection bias (B) on allocation concealment, performance bias (C), and detection bias (D) were identified as “unclear risk.” All experimental data included in articles were complete, so the attrition bias (E) and reporting bias (F) were considered to be low for 22 trials. There was insufficient information to assess the existence of other significant risk of bias, so other bias (G) of included trials were determined as “unclear risk.”

### 3.3. Outcome Measures with Subgroup Analysis

#### 3.3.1. Total Efficacy Rate of KFX Combined with PPI versus PPI Alone

**Figure 3:**
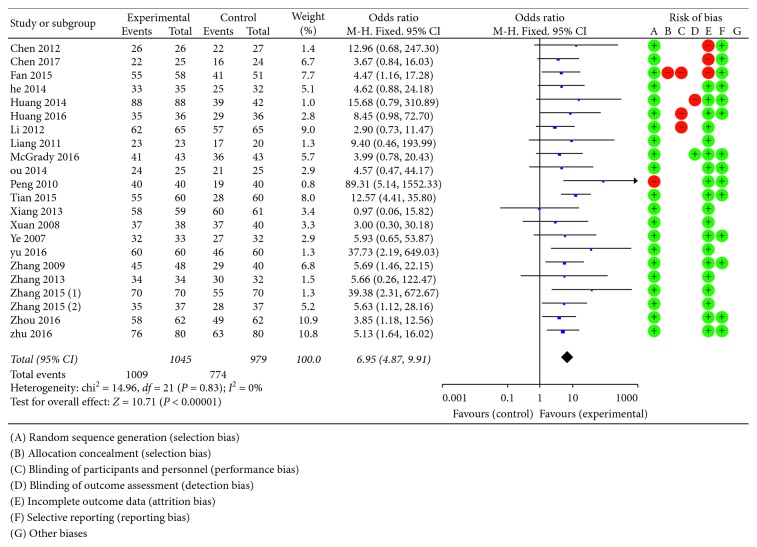
Forest plot of the total efficacy rate in patients treated with KFX + PPI and PPI alone and risk of bias. *I*^2^ and P are the criteria for the heterogeneity test; ◆, pooled odds ratio; —■—, odds ratio; and 95% CI. Quality assessment was conducted by Review Manager 5.3 according to Cochrane Handbook for Systematic Reviews of Interventions version 5.1.0. Red circle, high risk of bias; green circle, low risk of bias; and blank, unclear risk of bias.

**Figure 4:**
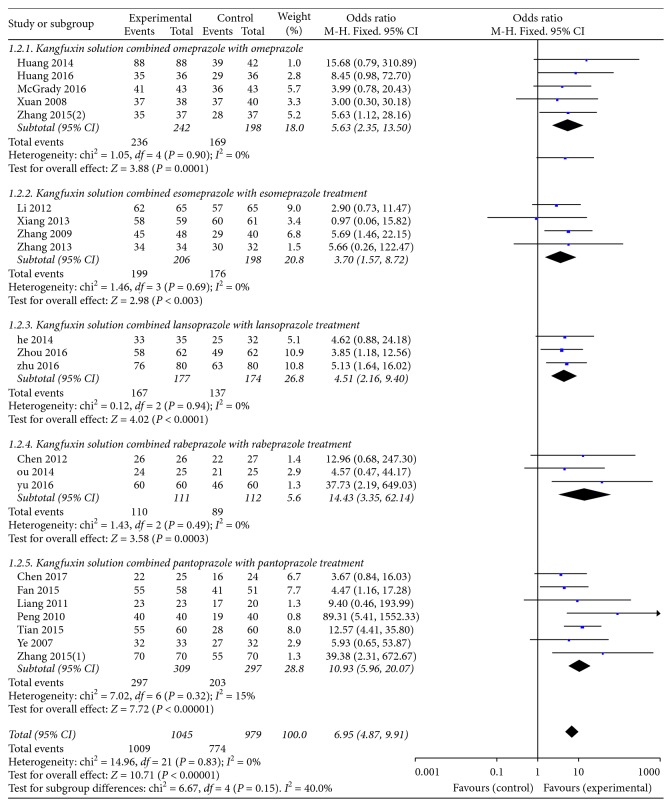
Forest plot of the total efficacy rate in patients treated with KFX + different PPI and PPI alone. *I*^2^ and *P* are the criteria for the heterogeneity test; ◆, pooled odds ratio; —■—, odds ratio; and 95% CI.

Total efficacy rate generally consists of grade of 6 clinical symptoms as follows: epigastric pain, belching, acid regurgitation, heartburn, satiety, nausea, and vomiting. Mark was given by assessing the severity and frequency of each item. For severity assessment, 0 points means asymptomatic, 1 point means symptoms were mild, 2 points between 1 and 3, and 3 points means symptoms were unbearable. For judgment of frequency, 0 points means asymptomatic, 1 point means the symptom have onset every 3∼4 days while 2 points means every 2 days, and 3 points means each day. The aggregate score was further divided into three levels: excellent, efficacious, and inefficient. All studies reported the total efficacy rate. A meta-analysis of these trials using a fixed-effect model demonstrated that KFX combined with PPI treatment significantly improved the total efficacy rate in the treatment of gastric ulcers (OR = 6.95, 95% CI: 4.87, 9.91; *P* < 0.00001). There was no statistically significant heterogeneity among individual trials (*P*=0.83, *I*^2^ = 0%). Risk of bias of each study is also listed (Figure 3). Further investigation was taken to explore the effectiveness of KFX combined with different PPIs for treating gastric ulcers. The total efficacy rate of KFX combined with omeprazole was significantly improved compared to omeprazole treatment alone (OR = 5.63, 95% CI: 2.35, 13.50; *P*=0.0001). No statistically significant heterogeneity was found among individual studies (*P*=0.90, *I*^2^ = 0%). Same results were found for the other PPIs as follows: KFX combined with esomeprazole (OR = 3.70, 95% CI: 1.57, 8.72; *P*=0.003), heterogeneity (*P*=0.69, *I*^2^ = 0%). KFX combined with lansoprazole (OR = 4.51, 95% CI: 2.16, 9.04; *P* < 0.0001), heterogeneity (*P*=0.94, *I*^2^ = 0%). KFX combined with rabeprazole (OR = 14.43, 95% CI: 3.35, 62.14; *P*=0.0003), heterogeneity (*P*=0.49, *I*^2^ = 0%). KFX combined with pantoprazole (OR = 10.93, 95% CI: 5.96, 20.07; *P* < 0.00001), heterogeneity (*P*=0.32, *I*^2^ = 15%). All meta-analyses above were analyzed using a fixed-effect model (Figure 4).

#### 3.3.2. KFX Combined Different PPIs on Efficacy Rate of Gastroscopy versus PPI Alone

**Figure 5:**
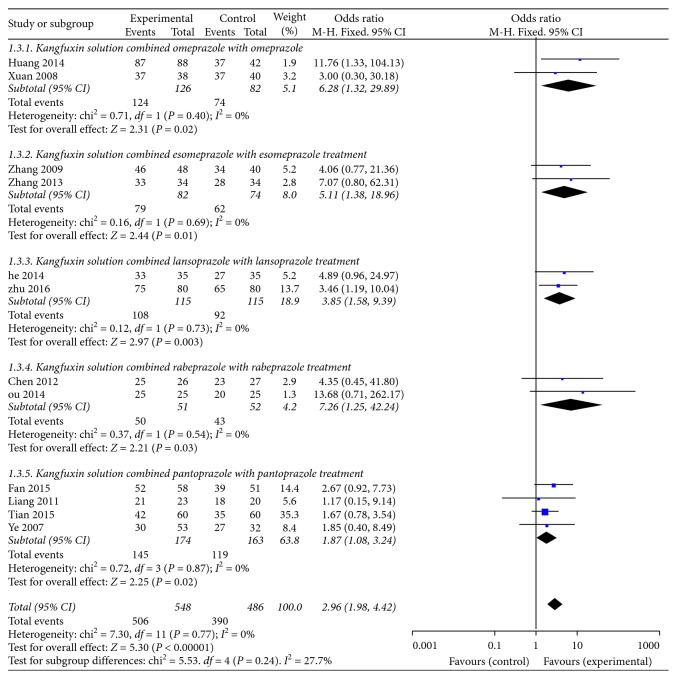
Forest plot of the efficacy rate of gastroscopy in patients treated with KFX + different PPI and PPI alone. *I*^2^ and *P* are the criteria for the heterogeneity test; ◆, pooled odds ratio; —■—, odds ratio; and 95% CI.

Criteria for judging the efficacy rate of gastroscopy were set as follows: clinical recovery was defined as that inflammation surrounding ulcer disappeared or the ulcer was scar over; the excellent effectiveness was identified as that ulcer disappeared but inflammation still existed; the efficacious effectiveness was defined as that the area of ulcer narrowed more than 50% or only a small amount of moss film attached to ulcer; and inefficient effect was that the area of ulcer narrowed less than 50% or no obvious change observed compared to prior treatment. 12 of 15 articles [22–24, 27, 28, 30–32, 34, 35, 37, 38] provided the efficacy rate of gastroscopy properly. A fixed-effect model demonstrated that KFX combined with PPI therapy significantly improved the efficacy rate of gastroscopy (OR = 2.96, 95% CI: 1.98, 4.42; *P* < 0.00001). No statistically significant heterogeneity was found among individual studies (*P*=0.77, *I*^2^ = 0%). 2 trials [24, 27] provided the combination of KFX and omeprazole treatment versus omeprazole alone; a fixed-effect model meta-analysis demonstrated that the combination significantly improved the efficacy rate of gastroscopy (OR = 6.28, 95% CI: 1.32, 28.89; *P*=0.02) with heterogeneity (*P*=0.40, *I*^2^ = 0%). 2 studies [22, 23] reported the combination of KFX and esomeprazole treatment (OR = 5.11, 95% CI: 1.38, 18.96; *P*=0.01) with heterogeneity (*P*=0.69, *I*^2^ = 0%). 2 studies [28, 30] reported the combination of KFX and lansoprazole treatment (OR = 3.85, 95% CI: 1.58, 9.39; *P*=0.003) with heterogeneity (*P*=0.73, *I*^2^ = 0%). 2 studies [31, 32] reported the combination of KFX and rabeprazole treatment (OR = 7.26, 95% CI: 1.25, 42.24; *P*=0.03) with heterogeneity (*P*=0.54, *I*^2^ = 0%). 4 studies [34, 35, 37, 38] reported the combination of KFX and pantoprazole treatment (OR = 1.87, 95% CI: 1.08, 3.24; *P*=0.02) with heterogeneity (*P*=0.87, *I*^2^ = 0%). A fixed-effect model was applied to finish the above-mentioned meta-analysis (Figure 5).

#### 3.3.3. Adverse Events

**Figure 6:**
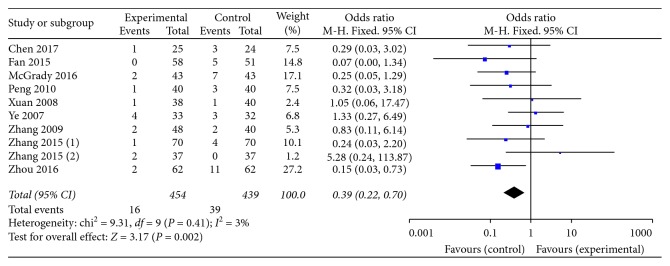
Forest plot of adverse events in patients treated with KFX + PPI and PPI alone. *I*^2^ and *P* are the criteria for the heterogeneity test; ◆, pooled odds ratio; —■—, odds ratio; and 95% CI.

Ten trials provided descriptions on adverse events generally including nausea, diarrhea, headache, constipation, rash, insomnia, dizziness, and bellyache (Table 2). A fixed-effect model analysis certified that the combination of KFX and PPI treatment reduced clinical adverse events significantly (OR = 0.39, 95% CI: 0.22, 0.70; *P*=0.002). No statistically significant heterogeneity was found among individual studies (*P*=0.41, *I*^2^ = 3%; Figure 6).

#### 3.3.4. Inflammatory Cytokines

**Figure 7:**
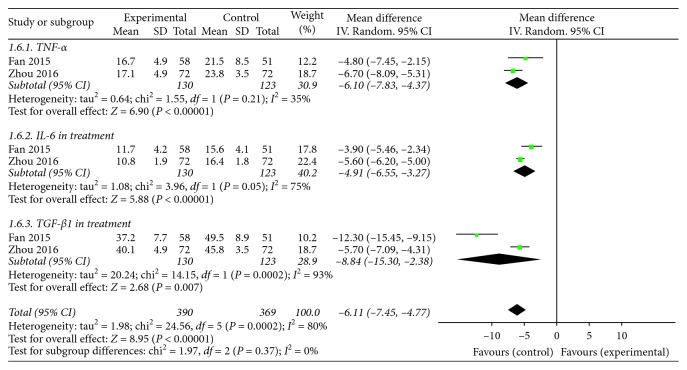
Forest plot of inflammation cytokines in patients treated with KFX + PPI and PPI alone. *I*^2^ and *P* are the criteria for the heterogeneity test; ◆, pooled mean difference; —■—, mean difference; and 95% CI.

Two trials [29, 34] reported the anti-inflammatory effects of KFX combined with PPI therapy versus PPI treatment alone. The serum contents of TNF-*α*, IL-6, and TGF-*β*1 were the common indices provided by the 2 studies. The pooled analysis (using a random-effect model) demonstrated that KFX combined with PPI treatment significantly relieved the inflammation of patients compared to PPI therapy alone (MD = −6.11, 95% CI: −7.45, −4.77; *P* < 0.00001). Statistically significant heterogeneity was observed among individual studies (*P*=0.0002, *I*^2^ = 80%). The further investigation was taken in subgroups. Combination treatment significantly reduced the serum content of TNF-*α* (MD = −6.10, 95% CI: −7.83, −4.37; *P* < 0.00001). No statistically significant heterogeneity was observed among individual studies (*P*=0.21, *I*^2^ = 35%). Influence of combination on IL-6 was reported as (MD = −4.91, 95% CI: −6.55, −3.27; *P* < 0.00001). Statistically significant heterogeneity (*P*=0.05, *I*^2^ = 75%) was observed among individual studies. Influence of combination on TGF-*β*1 was provided as (MD = −8.84, 95% CI: −15.30, −2.38; *P* < 0.007) with heterogeneity (*P*=0.0002, *I*^2^ = 93%; Figure 7). In consideration of the 2 trials reported KFX combined with lansoprazole and pantoprazole separately, the significant heterogeneity may be mainly generated by the different clinical treatments.

#### 3.3.5. T-Lymphocyte Subsets

**Figure 8:**
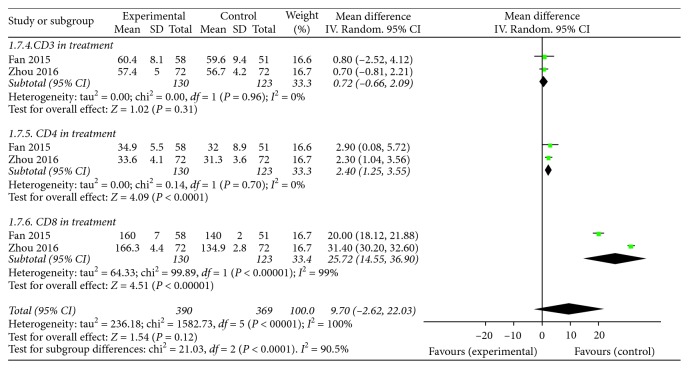
Forest plot of T-lymphocyte subsets in patients treated with KFX + PPI and PPI alone. *I*^2^ and *P* are the criterion for the heterogeneity test; ◆, pooled mean difference; —■—, mean difference; and 95% CI.

T-lymphocyte subset, which is the crucial index of immune function, was provided in 2 studies [29, 34]. There was heterogeneity in the index of CD8. Therefore, a random-effect model was used. There was no heterogeneity in the indices of CD3 and CD4; the fixed-effect model was thus used. The MD with 95% CI of serum CD3, CD4, and CD8 levels were (MD = 0.72, 95% CI: −0.66, 2.09; *P*=0.31), (MD = 2.40, 95% CI: 1.25, 3.55; *P* < 0.0001), and (MD = 25.72, 95% CI: 14.55, 36.90; *P* < 0.00001), respectively. There was no difference between the experimental group and control group (*P*=0.12; Figure 8).

#### 3.3.6. Recurrence Rate

**Figure 9:**
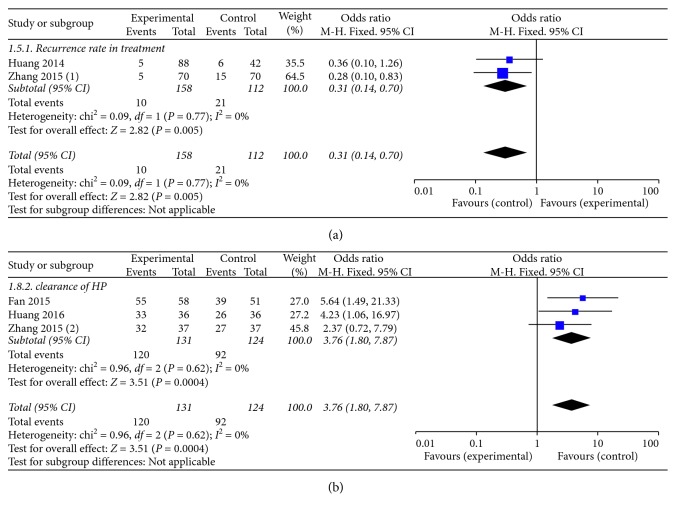
Forest plot of (a) the recurrence rate and (b) the clearance of HP in patients treated with KFX + PPI and PPI alone. *I*^2^ and *P* are the criteria for the heterogeneity test; ◆, pooled odds ratio; —■—, odds ratio; and 95% CI.

Two trials [24, 39] reported the recurrence rate in treatment. A meta-analysis (using a fixed-effect model) demonstrated that KFX combined with PPI significantly reduced the recurrence rate compared to PPI therapy alone (OR = 0.31, 95% CI: 0.14, 0.70; *P*=0.005). No statistically significant heterogeneity was found among individual studies (*P*=0.77, *I*^2^ = 0%; Figure 9(a)).

#### 3.3.7. Clearance Rate of HP

*Helicobacter Pylori* (HP) was thought to be the mainly inducing factor of GU. 3 studies [25, 34, 39] provided the clearance rate of HP in clinical treatment. A fixed-effect model analysis proved that the combination of KFX and PPI treatment enhanced the clearance of HP significantly (OR = 3.76, 95% CI: 1.80, 7.87; *P*=0.0004). No statistically significant heterogeneity was found among individual studies (*P*=0.62, *I*^2^ = 0%; Figure 9(b)).

#### 3.3.8. Publication Bias

**Figure 10:**
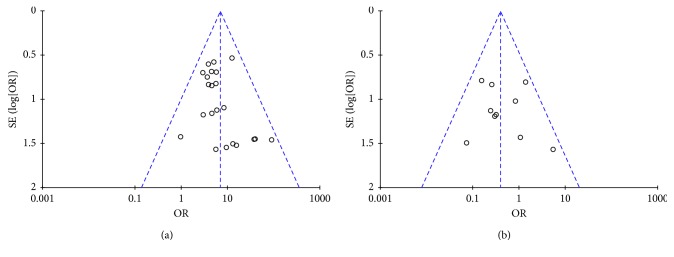
Funnel plot for the publication bias: (a) total efficacy rate; (b) adverse events.

A funnel plot was used to express publication bias. When the indices were provided by more than 9 cases, the publication was explored. In the present study, the funnel plot of combination of KFX and PPIs versus PPIs therapy alone on total efficacy rate and adverse events was applied. The plots were generally symmetric, suggesting that there was no obvious publication bias (Figures 10(a) and 10(b)).

## 4. Discussion

“No acid, no ulcer” said by Schwartz indicated that excessive gastric acid secretion and GU are highly related. PPIs are the very class of medicines that are invited to decrease gastric acid secretion via inhibiting the H^+^/K^+^-ATP pump of the parietal cell. United States Food and Drug Administration (FDA) approved the first PPI omeprazole in 1980s. Today, 5 other PPIs are also employed to treat a variety of acid-related conditions such as duodenal ulcers, gastric ulcers, and *Helicobacter pylori* eradication. PPIs are widely accepted to be the most effective treatment for symptom relief of gastro-oesophageal reflux [43–45]. Due to its good effect and the growing number of PPIs available over-the-counter, market of PPIs booms rapidly. However, accompanied by continuous appearance of adverse effects we discussed earlier, some scholars expressed concern about unnecessary use of PPIs which is so high in their latest review [46]. The huge market sales in China suggest that the consumption of PPIs is very enormous [5]. Scholars in China also put an immense concern on overmedication of PPIs [11]. Actions should be taken to pull back PPIs to the road of rational drug use.

*Periplaneta americana* also known as cockroach is an insect of *Blattodea* recorded most early in “Sheng Nong's herbal classic.” It was classified as middle grade. CFDA has approved 4 patent drugs including Kangfuxin liquid, Ganlong capsule, Xiaozheng Yigan tablets, and Xinmailong injection all extracted from *Periplaneta americana* but aimed at different diseases. Our previous study has demonstrated that extract of *Periplaneta americana* had good protective effects on GU in animal models [47, 48]. Recent meta-analysis conformed that KFX combined with PPIs was superior to PPIs alone in the treatment of GU in total efficacy rate [17, 18]. Here, in this paper, we further affirmed these findings and report an extended result. Compared to PPIs therapy alone, combination with KFX exerted significant improvement in total efficacy rate and efficacy rate of gastroscopy (*P* < 0.00001, *P* < 0.00001, respectively). The combination also reduced the adverse events and the recurrence rate (*P*=0.002, *P*=0.005, respectively). It was also associated with a significant enhancement of HP clearance (*P*=0.0004). The efficacy may be associated with relieving the inflammation of patients (*P* < 0.00001) but not boosting immunity (*P*=0.12). Conclusions on recurrence rate, clearance of HP, inflammatory cytokines, and T-lymphocyte subsets are based on only two or three small-sample studies which should be treated with caution.

We also performed a subgroup analysis on KFX combined with different PPIs on total efficacy rate. No obvious difference was found between PPIs though the second generation PPIs including esomeprazole and rabeprazole was claimed for having better affects. Firstly, we apologized for the limitations of our work, but we also found an explanation in the methodologies of trials included. Most of the trials put a final assessment on improvement of total efficacy rate instead of interval evaluation that may lead to a different conclusion.

Three articles [27, 40, 42] reported anaphylaxis, such as rash of PPIs, which reminds us that we should also pay attention to adverse reactions in short-term medication. The US FDA issued a warning on all PPIs in 2010 stating that patients should use the lowest dose and shortest duration of PPI therapy due to the increased risks [46]. Combination with TCM should be taken into consideration for effect-enhancing and/or side effect-mitigating.

The current research is not registered, and there may be a small offset, but the meta-analysis was produced strictly in accordance with the process of systematic review. However, due to the low quality of clinic trials cited, the accuracy of the results in this paper will be affected to some extent and should be handled cautiously.

## 5. Conclusion

These findings indicate that the combination of KFX and PPIs may significantly improve the total efficacy rate and efficacy rate of gastroscopy and reduce clinical adverse events. Due to the small sample size and limitations of this study, we sound a cautious note that KFX combined with PPIs may relieve the inflammation of patients, boost immunity, reduce recurrence rate, and enhance the clearance of HP. However, our findings must be handled with care because of the low quality of clinic trials cited. Other rigorous and large-scale RCTs are in need to confirm these results.

## Figures and Tables

**Table 1:** Characteristics of eligible studies.

Author and published year (references)	Cases T/C	Diagnostic standard	Age (years), range, mean	Sex (male/female)
Li, 2012 [20]	65/65	DTDUD (2008)	T: 24–69, 43.2	T: 45/20
			C: 22–70, 41.3	C: 47/18
Xiang, 2013 [21]	59/61	GPCRTPU	T: 39.4	NR
			C: 40.7	
Zhang, 2009 [22]	25/24	DDD (2006)	T: 27–52, 38	T: 16/9
			C: 29–58, 39	C: 13/11
Zhang, 2013 [23]	34/32	Gastroscopy	T: 24–68, 34	T: 20/14
			C: 20–65, 32	C: 19/13
Huang, 2014 [24]	88/42	Gastroscopy	T: 18–60, NR	NR
			C: 18–60, NR	
Huang, 2016 [25]	36/36	DTDUD (2013)	T: 29–76, 35.6	T: 21/15
			C: 28–74, 35.5	C: 20/16
McGrady, 2016 [26]	43/43	DTDUD (2013)	T: 20–65, 35.6	T: 28/15
			C: 20–66, 36.1	C: 25/18
Xuan, 2008 [27]	38/40	Gastroscopy	T: 20–65, NR	NR
			C: 20–65, NR	
Zhu, 2016 [28]	80/80	Gastroscopy	T: 23–67, 38.9	T: 52/28
			C: 19–65, 39.4	C: 49/31
Zhou, 2016 [29]	62/62	Gastroscopy	T: 47.2	T: 37//25
			C: 46.4	C: 36/26
He, 2014 [30]	35/32	Gastroscopy	T: 18–72, 43	T: 23/12
			C: 20–75, 45	C: 22/10
Chen, 2012 [31]	26/27	Gastroscopy	24–78, 61.5	28/25
Ou, 2014 [32]	25/25	Gastroscopy	25–60, 45	26/24
Yu, 2016 [33]	60/60	DTDUD (2013)	18–70, NR	T: 40/20
				C: 42/18
Chen, 2017 [15]	25/24	DTDUD (2016)	T: 27–67, 44.5	T: 14/11
			C: 26–68, 45.2	C: 15/9
Fan, 2015 [34]	58/51	Gastroscopy	T: 43.8	T: 29/29
			C: 42.6	C: 30/21
Liang, 2011 [35]	23/20	Gastroscopy	T: 31–77, 49.1	T: 15/8
			C: 27–72, 46.6	C: 11/9
Peng, 2010 [36]	40/40	Gastroscopy	T: 19–43, 33.5	T: 26/14
			C: 19–42, 32.4	C: 28/12
Tian, 2015 [37]	60/60	PCDTDD (2005)	T: 17–67, 34.5	T: 42/18
			C: 19–71, 33.5	C: 40/20
Ye, 2007 [38]	33/32	Gastroscopy	T: 20–63, 38.5	NR
			C: 20–65, 39.8	
Zhang, 2015 [39]	70/70	NR	T: 23–72, 45.2	T: 42/28
			C: 25–70, 43.3	C: 43/27
Zhang, 2015 [40]	37/37	Gastroscopy	T: 29–72, 34.7	T: 26/11
			C: 28–75, 35.8	C: 24/13

T, trial group; C, control group; NR, no report.

**Table 2:** Intervention characteristics of included studies.

Study ID (name, year)	Intervention		Duration/follow-up	Adverse events	Outcome measures
	Trial group	Control group (essential treatment)			
Li, 2012 [20]	KFX, 10 mL, TID + essential treatment	Esomeprazole, 40 mg, QD, Po	4 weeks/NR	NR	Total efficacy rate, efficacy rate of gastroscopy
Xiang, 2013 [21]	KFX, 10 mL, TID + essential treatment	Esomeprazole, 40 mg, QD, Po	4 weeks/NR	NR	Total efficacy rate, efficacy rate of gastroscopy
Zhang, 2009 [22]	KFX, 10 mL, TID + essential treatment	Esomeprazole, 40 mg, QD, Po	4 weeks/NR	T: 2 cases nausea, 1 case diarrhea; C: 2 cases nausea	Total efficacy rate, efficacy rate of gastroscopy
Zhang, 2013 [23]	KFX, 10 mL, TID + essential treatment	Esomeprazole, 20 mg, QD, Po	4 weeks/NR	NR	Total efficacy rate, efficacy rate of gastroscopy
Huang, 2014 [24]	KFX, 10 mL, TID + essential treatment	Omeprazole, 20 mg, BID, Po	4 weeks/1 year	NR	Total efficacy rate, efficacy rate of gastroscopy, recurrence rate
Huang, 2016 [25]	KFX, 10 mL, TID + omeprazole, 20 mg, QD, Po	Omeprazole, 20 mg, BID, Po	T: 28 days/NR C: 14 days/NR	NR	Total efficacy rate, clearance rate of HP
McGrady, 2016 [26]	KFX, 10 mL, TID + essential treatment	Omeprazole, 20 mg, BID, Po	4 weeks/NR	T: 1 case headache, 1 case nausea; C: 3 cases headache, 2 cases nausea, 2 cases constipation	Total efficacy rate
Xuan, 2008 [27]	KFX, 10 mL, TID + omeprazole, 20 mg, QD, Po	Omeprazole, 20 mg, BID, Po	4 weeks/NR	T: 1 case rash over axillae; C: 1 case insomnia	Total efficacy rate, efficacy rate of gastroscopy
Zhu, 2016 [28]	KFX, 10 mL, TID + essential treatment	Lansoprazole, 30 mg, QD, Po	2 weeks/NR	NO	Total efficacy rate, efficacy rate of gastroscopy
Zhou, 2016 [29]	KFX, 10 mL, TID + essential treatment	Lansoprazole, 30 mg, QD, Po	4 weeks/NR	T: 2 cases dizziness and diarrhea; C: 11 cases nausea and diarrhea	Total efficacy rate, efficacy rate of gastroscopy, inflammatory cytokines, T-lymphocyte subsets
He, 2014 [30]	KFX, 10 mL, TID + essential treatment	Lansoprazole, 30 mg, QD, Po	4 weeks/NR	NO	Total efficacy rate, efficacy rate of gastroscopy
Chen, 2012 [31]	KFX, 10 mL, TID + essential treatment	Rabeprazole, 10 mg, QD, Po	4 weeks/NR	NR	Total efficacy rate, efficacy rate of gastroscopy
Ou, 2014 [32]	KFX, 10 mL, TID + essential treatment	Rabeprazole, 10 mg, QD, Po	5 weeks/NR	NR	Total efficacy rate, efficacy rate of gastroscopy
Yu, 2016 [33]	KFX, 10 mL, TID + essential treatment	Rabeprazole, 20 mg, QD, Po	3 weeks/NR	NR	Total efficacy rate
Chen, 2017 [15]	KFX, 10 mL, TID + essential treatment	Pantoprazole, 40 mg, BID, Po	2 weeks/NR	T: 1 case rash; C: 2 cases nausea, 1 case rash	Total efficacy rate
Fan, 2015 [34]	KFX, 10 mL, TID + essential treatment	Pantoprazole, 40 mg, QD, Po	4 weeks/NR	T: NO; C: 5 cases diarrhea	Total efficacy rate, efficacy rate of gastroscopy, inflammatory cytokines, T-lymphocyte subsets, clearance rate of HP
Liang, 2011 [35]	KFX, 10 mL, TID + essential treatment	Pantoprazole, 40 mg, BID, intravenous drip	2 weeks/NR	NR	Total efficacy rate, efficacy rate of gastroscopy
Peng, 2010 [36]	KFX, 10 mL, TID + essential treatment	Pantoprazole, 40 mg, BID, Po	10 days/NR	T: 1 case diarrhea; C: 2 cases diarrheoa, 1 case bellyache	Total efficacy rate
Tian, 2015 [37]	KFX, 10 mL, QD + essential treatment	Pantoprazole, 40 mg, QD, Po	1 month/NR	NR	Total efficacy rate, efficacy rate of gastroscopy
Ye, 2007 [38]	KFX, 10 mL, TID + essential treatment	Pantoprazole, 40 mg, QD, Po	4 weeks/NR	T: 4 cases digestive symptoms; C: 3 cases digestive symptoms	Total efficacy rate, efficacy rate of gastroscopy
Zhang, 2015 [39]	KFX, 10 mL, TID + essential treatment	Pantoprazole, 40 mg, BID, intravenous drip	1 week/half a year	T: 1 case diarrhea; C: 2 cases diarrhea, 1 case headache, 1 case rash	Total efficacy rate, recurrence rate
Zhang, 2015 [40]	KFX, 10 mL, TID + omeprazole, 20 mg, QD, Po	Omeprazole, 20 mg, BID, Po	T: 4 weeks/NR C: 2 weeks/NR	T: 2 case diarrhea; C: NO	Total efficacy rate, clearance rate of HP

QD, once a day; BID, twice a day; TID, three times a day; KFX, Kangfuxin Solution; Po, oral administration; HP, *Helicobacter pylori*; NR, no report.

## Supplementary Materials

Supplementary Materials Preferred Reporting Items for Systematic Reviews and Meta-Analyses (PRISMA) guidelines containing 27 checklist items of the PRISMA statement pertain to the content of a systematic review and meta-analysis, which include the title, abstract, methods, results, discussion, and funding.
